# Spatiotemporal Patterns of Ecosystem Service Value Changes and Their Coordination with Economic Development: A Case Study of the Yellow River Basin, China

**DOI:** 10.3390/ijerph17228474

**Published:** 2020-11-16

**Authors:** Aijun Guo, Yongnian Zhang, Fanglei Zhong, Daiwei Jiang

**Affiliations:** School of Economics, Lanzhou University, Lanzhou 730000, China; guoaj@lzu.edu.cn (A.G.); zhangyn2019@lzu.edu.cn (Y.Z.); jiangdw18@lzu.edu.cn (D.J.)

**Keywords:** ecosystem service values, spatiotemporal analysis, geospatial data, nonparametric test, Yellow River

## Abstract

By integrating multiple remote sensing data sources this study accurately assesses the spatiotemporal characteristics of changes in ecosystem service values (ESVs) in the Yellow River Basin from 2000 to 2015 through Theil-Sen median trend analysis and the Mann-Kendall test. The stability and continuity of the ESVs were comprehensively characterized using coefficients of variation and the Hurst exponent. The degree of coherence between ESVs and economic growth (represented by gross domestic product GDP) on the same temporal and spatial scales was analyzed using ecological-economic coordination (EEC) models. The results show that (1) from 2001 to 2015 the total ESV and the ESV per unit area in the Yellow River Basin generally showed a U-shaped pattern (decreasing slightly then increasing rapidly). (2) The areas with increasing ESVs made up approximately 55.6% of the total area of the river basin. The areas with a decreasing pattern were mainly in the west and north of the Yellow River Basin. (3) The stability and continuity of the ESVs showed a clustered, compact distribution. (4) The most common level of EEC was slightly uncoordinated followed by slightly coordinated and highly coordinated. The proportion of coordinated areas was relatively higher in cultivated land and the lowest in built-up land.

## 1. Introduction

Ecosystem services are the contributions that Nature makes to human well-being [1,2,3]. The transport and processing of materials, information, and energy among all components within the ecosystem provide physical material resources and intangible ecosystem service values (ESVs) to humans [4,5]. Linking Nature and humans together, human production activities integrate ecosystems and economic systems into an organic whole [6,7]. Since the late middle of the 20th century, human society-economic development has had a significant impact on land use change. This change has significantly affected ecosystem services functions, leading to the weakening of ESV [8,9]. The loss and degradation of ESV has aroused widespread concern on the coordinated development of ecological-economic system [1,10]. The concept of ecological-economic coordination (EEC) emphasizes that the two systems can achieve overall sustainable development through interaction and mutual influence [11,12]. Economic development must be achieved in conjunction with the development and utilization of local resources and the protection of ecological environment. The degree of coordination is crucial for comprehensive and sustainable development in a region [13,14,15]. Therefore, there is an urgent need to quantify the value of ecosystem services and the level of EEC to achieve sustainable ecological and economic development.

ESV evaluation is a research hotspot in ecology and source environmental economics, which was first conceptualized in the 1970s [16,17]. Since 1990, ESV evaluations have been widely performed and studied at different spatial and temporal scales [18,19]. Sufficient evaluations of ESV have become a reliable basis for ecosystem asset management, ecological compensation, and the paid use of ecosystem services. It is an effective way to achieve regional and even global sustainable development [20,21]. From the view of research methods, two ways are popular for ESV evaluations, including one based on the unit price of ecosystem service products and another based on the unit area’s equivalent value factor [22]. The former mainly utilizes ecological models to quantify ecosystem processes and functions and then estimates the value of ecosystem services using the direct market method, alternative market method or simulated market value method [8,23,24]. This method is mainly applied in cases such as small-scale areas or individual ecosystems due to its high data requirements and complex calculations [25,26]. The second method takes the initial ESV per unit area as input to derive the comprehensive ESV calculations [18,20]. This method is relatively simple and applicable to ESV assessment in cases of land use change and is widely used in cases with large-scale areas [22,27,28].

The method based on the equivalent value factor of unit area can directly reflect the change in ESVs caused by changes in land use status from human activities. It has been widely used in large-scale research in China [28,29]. Generally, current research on ESVs based on the equivalent value factor of unit area largely suffers from the following shortcomings. (a) The static estimation method based on the unit area value for the total ESV does not take area and quality differences among ecosystems in into account, which may lead to errors in regional estimation results. (b) Although there are many studies on the spatiotemporal patterns and characteristics of ESVs, they are short on discussions of future development trends and optimized solutions. (c) Few models have been constructed to dynamically assess the complex relationships between the impact of ecosystems on human well-being and social-economic growth. (d) Most research has mainly focused on ESVs on a large scale, such as at the national level, whereas studies at the watershed level are still rare.

The Yellow River is credited as the mother river of the Chinese nation and has played and continues to play an important role in China’s socioeconomic development and the ecological security maintenance. In September 2019, the Ecological Conservation and High-quality Development plan was established as a major national strategy. This plan is an important measure for China to develop an ecological civilization and promote high-quality regional economic development in the new era. Accurate assessment of ESVs is the key to supporting the construction of an ecological civilization. There is a lack of research on the ESV of the Yellow River Basin, so it is necessary to focus more attention and effort on this issue. This study focuses on spatial patterns and a refined scale as the starting points to analyze the ESVS and the characteristics of EEC in the Yellow River Basin from a grid perspective. The main objectives are as follows: (a) In combination with spatial analysis, this work utilizes multisource remote sensing data to build an ESV assessment model. Based on a comprehensive consideration of ecosystem types, current statuses, and spatial differences, quantitative and accurate assessment of the ESVs in the Yellow River Basin is conducted. (b) The temporal and spatial change trends of the ESVs in the Yellow River Basin from 2000 to 2015 are measured, and the predictions of the future development trends are established. (c) A coordinated development model of spatial ecosystem relationships is established at the grid scale to dynamically evaluate the complex relationships among ecosystems, human well-being, and social-economic growth. The comprehensive study in this paper can help to deepen the understanding of the processes and pattern characteristics of ESVs at large basin scales. This paper also aims to provide a scientific basis for the proposal of potential policies for ecological protection and high-quality economic development in the Yellow River Basin.

## 2. Study Area

**Figure 1:**
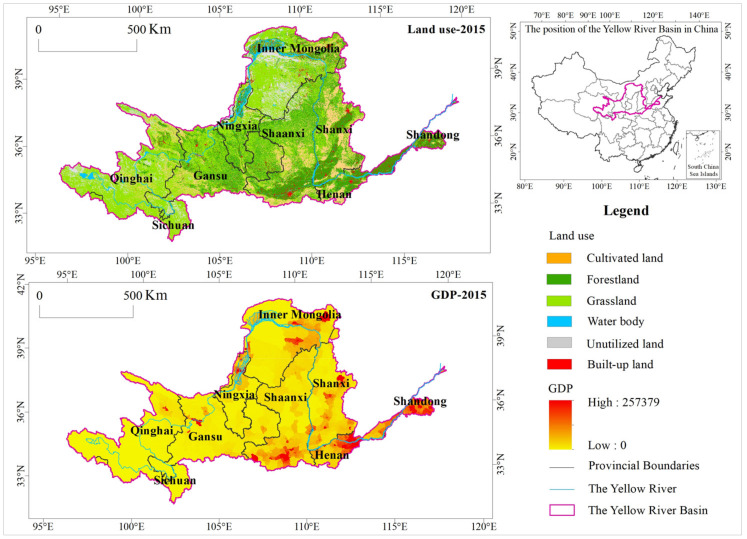
Location of the study area.

The Yellow River Basin is located between 96°53′–119°05′ E and 32°10′–41°50′ N. From west to east, it includes nine provinces and autonomous regions: Qinghai, Sichuan, Gansu, Ningxia, Inner Mongolia, Shaanxi, Shanxi, Henan, and Shandong. In 2015, the total GDP of these nine provinces was 19,123.8 billion yuan. The river ultimately drains into the Bohai Sea, and the area of the basin is approximately 79.5 × 10^4^ km^2^ [30] (Figure 1). The Yellow River Basin mainly belongs to the southern temperate zone, mid-latitude temperate zone, and the plateau climate zone. Precipitation is unevenly distributed, and the mean annual precipitation is 476 mm [31]. The land use types are mainly forestland, grassland and cultivated land, accounting for 25.8%, 47.4% and 13.4% of the total area of the basin, respectively, with some regional differences. Forestland is mainly distributed in the southeast of the basin, and grassland is mainly distributed in the central and western regions [32]. Additionally, The Yellow River Basin serves as an ecological corridor that links the Qinghai-Tibet Plateau, the Loess Plateau, and the North China Plain. A number of national parks and national key ecological function areas, such as the Three Rivers’ Headstream Region and Qilian Mountains, are located in the basin area [33]. Their functions of windbreak, soil retention, water conservation, and biodiversity provide critical support for the regional ecological security maintenance and environmental protection [34].

## 3. Materials and Methods

### 3.1. Data Collection

Normalized difference vegetation index (NDVI) data with a spatial resolution of 1 km were derived from Geospatial Data Cloud [35]. Monthly data were synthesized into annual data using the maximum value synthesis method. Net primary productivity (NPP) data were acquired from the MOD17A3 product provided by the Numerical Terradynamic Simulation Group, College of Forestry & Conservation, University of Montana [36]. The data were calculated based on the BIOME-BGC model with a spatial resolution of 1 km. Land use and spatial gross domestic product (GDP) data came from the Data Center for Resources and Environmental Sciences of the Chinese Academy of Sciences [37]. Land use data were interpreted and classified using Landsat remote sensing images. Land use types were classified as forestland, grassland, cultivated land, waterbody, urban and rural built-up land, and unutilized land, with a spatial resolution of 1 km. Spatialized GDP grid data came from rasterization of county GDP data in conjunction with night light levels, densities of residential communities, and land use types.

The classification and initial per unit area values of ecosystems service are based on the research results by Xie et al. [38]. Ecosystem services include regulating services (e.g., gas regulation, climate regulation, water conservation waste treatment), material provisioning services (e.g., food production, raw material), support service (e.g., soil formation and protection, biodiversity conservation) and cultural services (e.g., entertainment culture) [38,39]. The per unit area ESVs of different land covers have been obtained through 200 ecologists’ comprehensive scorings. In this paper, we adopted the estimated per-square-kilometer ESVs of the six land covers in China (Table 1). [39].

### 3.2. Valuation of ESV

ESVs are valued by using certain accounting standards to estimate the sum of human well-being that can be measured in money provided by ecosystem services at a certain time. The calculation formula is [3,29]:

(1) $$ESV=\sum_{c=1}^{m}ESV_{c}\;$$

where *ESV* represents the ecosystem service values, *c* represents the type of the constituent unit of the ecosystem, and *ESV_C_* represents the ESV of the *c* type of ecosystem unit, which is calculated as follows:

(2) $$ESV_{c}=\sum_{i=1}^{n}\sum_{j=1}^{m}R_{ij}\times V_{ci}\times S_{ij}$$

where *i* represents the *i* type of ecosystem service function of the *c* ecosystem unit, *V_ci_* represents the value equivalent factor of the *i* type ecosystem service of the *c* ecosystem unit, *j* represents the total number of patches in a specific area, *S_ij_* indicates the area size of each patch, *R_ij_* indicates the ecological adjustment parameters of *V_ci_* in different patches, which are usually the NPP and vegetation coverage (*f*) (NDVI) and represents the quality of the ecosystem. The formula for *R_ij_* is as follows [40]:

(3) $$R_{ij}=\left(NPP_{j}/NPP_{mean}+f_{j}/f_{mean}\right)/2$$

(4) $$f=\frac{NDVI-NDVI_{\min}}{NDVI_{\max}-NDVI_{\min}}$$

where *NPP_mean_* and *f_mean_* are the average values of NPP and vegetation coverage, respectively; *NPP_j_* and *f_j_* are the maximum and minimum values of the NPP and vegetation coverage of the *j* unit, respectively; and *NDVI_max_* and *NDVI_min_* are the maximum and minimum values, respectively, of the NDVI. It is necessary to note that the Yellow River Basin crosses nine provinces/autonomous regions in China from west to east. Their boundaries are based on natural divisions. When calculating the ESV data by region, the data included in this study were based on the Yellow River Basin areas.

### 3.3. Theil-Sen Median Trend Analysis and Mann-Kendall Test

The combination of Theil-Sen median trend analysis and the Mann-Kendall test can be used to examine the change characteristics of time-series ESVs from the spatial grid perspective. Theil-Sen median trend analysis is a robust, nonparametric statistical trend calculation method [41,42]. This method is not affected by data outliers and has a strong ability to avoid errors. The data do not need to conform to a certain distribution. It can scientifically and objectively reflect the evolutionary trend of the ESVs. The Theil-Sen median trend calculates the median of the slope of n (n − 1)/2 data combinations [43], and its calculation formula is:

(5) $$S_{ESV}=Median\left(\frac{ESV_{j}-ESV_{i}}{j-i}\right),\;2000\leq i<j\leq 2015$$

When *S_ESV_* > 0, this reflects an increasing trend of the time-series ESVs; otherwise, it reflects a decreasing trend of the time-series ESVs.

The calculation formula of the Mann-Kendall test is [44,45]:

Set $\left(ESV_{i}\right),\;i=2000,\;2005\;,\cdots \;2015$

(6) $$\mathrm{defines}\;\mathrm{the}\;Z\;\mathrm{statistics}\;\mathrm{as}\;Z=\left\{\begin{array}{c} \frac{S-1}{\sqrt{s\left(S\right)}}\;,\;S>0\\ \;0\;,\;S=0\\ \frac{S+1}{\sqrt{s\left(S\right)}}\;,\;S<0\; \end{array}\right.,\;\mathrm{where}\;S=\sum_{j=1}^{n-1}\sum_{i=j+1}^{n}sgn{\left(ESV_{j}-ESV_{i}\right)}_{\;}$$

(7) $$sgn\left(ESV_{j}-ESV_{i}\right)=\left\{\begin{array}{c} 1\;,\;\mathrm{ES}V_{j}-ESV_{i}>0\\ 0\;,\;\mathrm{ES}V_{j}-ESV_{i}=0\\ -1\;,\;\mathrm{ES}V_{j}-ESV_{i}<0 \end{array}\right.,s\left(S\right)=\frac{n\left(n-1\right)\left(2n+5\right)}{18}$$

where *ESV_j_* and *ESV_i_* are the ESV pixels in the *i* and *j* years, respectively, and *n* represents the time-series range of the study. The range of the Z statistic is (−∞, +∞), and we generally take the statistic with probability values *α* = 0.05 or *α* = 0.01, indicating that the time-series evolution trend of the ESVs is significant at the 95% or 99% confidence level, respectively.

The Sen trend estimator was superimposed with the |Z| value of the Mann-Kendall test to obtain the spatial trend of the grid unit ESV, and the calculation results were divided into four categories: significantly reduced (S_ESV_ < 0, |Z| > 1.65), nonsignificantly reduced (S_ESV_ < 0, |Z| < 1.65), nonsignificantly increased (S_ESV_ > 0, |Z| < 1.65), and significantly increased (S_ESV_ > 0, |Z| > 1.65).

### 3.4. Stability and Continuity Model

The coefficient of variation can reflect the degree of dispersion in the data [46,47]. Therefore, the coefficient of variation method was selected to evaluate the stability of the ESVs in time series. The calculation formula is:

(8) $$C_{ESV}=\frac{ST_{ESV}}{\bar{ESV}}$$

where *C_ESV_* is the coefficient of variation, *ST_ESV_* represents the standard deviation of the *ESV*, and $\bar{ESV}$ $\bar{}$ is the mean of the ESV. The greater the value of *C_ESV_* is, the more dispersed the data, the greater the degree of fluctuation, and the worse the stability of the ESVs. On the other hand, the more concentrated the distribution of the data is, the steadier the ESV.

The Hurst exponent is a method for distinguishing sustainability of time-series data [48,49]. The Hurst exponent was used to characterize the variation patterns of continuity in the ESVs. For time series {R_(t)_}, *t* = 1, 2, …, *n* and for any positive integer *τ*≥1, the mean series exists [41,50]:

(9) $${\bar{R}}_{\left(\tau \right)}=\frac{1}{\tau }\sum_{t=1}^{\tau }R_{\left(\tau \right)}\;\tau =1,2,\cdots \cdots \cdots ,n$$

(10) $$\mathrm{Cumulative}\;\mathrm{dispersion}:X_{\left(t,\tau \right)}=\sum_{t=1}^{t}(R_{\left(t\right)}-{\bar{R}}_{\left(\tau \right)})\;1\leq t\leq \tau$$

(11) $$\mathrm{Range}\;R_{\left(\tau \right)}=\max X_{\left(t,\tau \right)}-\min X_{\left(t,\tau \right)}\;\mathrm{where}\tau =\;1,\;2,\;\ldots ,n_{\;}$$

(12) $$\mathrm{Standard}\;\mathrm{deviation}\;S_{\left(\tau \right)}={\left[\frac{1}{\tau }\sum_{t=1}^{\tau }(R_{\left(t\right)}-R_{\left(\tau \right)}{)}^{2}\right]}^{\frac{1}{2}}\;\mathrm{where}\tau =\;1,\;2,\;\ldots ,n$$

Here, *R/S* = *R*(*τ*)/*S*(*τ*). If *R/S*∝*τ^H^*, then the Hurst phenomenon exists in the time series of the analysis, and the Hurst exponent H is obtained by least-squares fitting. Based on the value of H, the continuity characteristics of the value change of ESVs can be comprehensively judged. When 0.5 < H < 1, the future trend of ESVs is basically consistent with the past changes, and the consistency monotonically increases with an increase in H. When 0 < H < 0.5, the trend of ESVs is noncontinuous. When H = 0.5, the time series of ESVs is random and therefore does not have the characteristic of correlation.

Based on the changing trend of ESVs and the effective combination of the coefficient of variation and the Hurst exponent, the results were divided into eight categories: 1. continuous steady decline (S_ESV_ < 0, C_ESV_ < 0.5, 0.5 < H < 1), 2. continuous unsteady decline (S_ESV_ < 0, C_ESV_ < 0.5, 0.5 < H < 1), 3. noncontinuous steady increase (S_ESV_ > 0, C_ESV_ > 0.5, 0 < H < 0.5), 4. noncontinuous unsteady increase (S_ESV_ > 0, C_ESV_ < 0.5, 0 < H < 0.5), 5. noncontinuous steady decrease (S_ESV_ < 0, C_ESV_ > 0.5, 0 < H < 0.5), 6. noncontinuous unsteady decline (S_ESV_ < 0, C_ESV_ < 0.5, 0 < H < 0.5), 7. continuous steady increase (S_ESV_ > 0, C_ESV_ > 0.5, 0.5 < H < 1), and 8. continuous unsteady increase (S_ESV_ > 0, C_ESV_ < 0.5, 0.5 < H < 1).

### 3.5. Ecological-Economic Coordination Level Model

Based on the decoupling theory construct EEC index proposed [14,15]. EEC refers to the ratio of the change rate of ESV per unit area to the change rate of GDP per unit. The calculation formula is:

(13) $$EEC=\frac{ESV_{pr}}{GDP_{pr}}\;,\;\mathrm{where}\;GDP_{pr}=\frac{GDP_{pe}-GDP_{ps}}{GDP_{ps}},\;ESV_{pr}=\frac{ESV_{pe}-ESV_{ps}}{ESV_{ps}}$$

In the formula, *ESV_pr_* and *GDP_pr_* represent the change rates of the ESV and GDP, respectively, from the starting year to the ending year; *GDP_pe_* and *GDP_ps_* represent the GDP of the grid unit of the starting year and ending year, respectively; and *ESV_pe_* and *ESV_ps_* represent the ESV of the grid unit of the starting year and ending year, respectively.

Based on existing research and the various characteristics of the ESVs in the Yellow River Basin, the EEC is divided into the following eight levels [14,15]: EEC < −1 indicates highly uncoordinated areas, −1 < EEC < −0.6 indicates mostly uncoordinated areas, −0.6 <EEC < −0.4 indicates moderately uncoordinated areas, −0.4 < EEC < 0 indicates slightly uncoordinated areas, 0 < EEC < 0.4 indicates slightly coordinated areas, 0.4 < EEC < 0.6 indicates moderately coordinated areas, 0.6 < EEC < 1 indicates mostly coordinated areas, and EEC > 1 indicates highly coordinated areas.

On this basis, a filter with a 3 × 3 window was introduced. When three-quarters or five-eighths of the connected raster pixels in the window had the same value, the pixels were smoothed. The obtained results were included in iterations until the data were finally steady. The data-smoothing process was used because this process aggregates the relatively discrete EEC levels to maximize the emphasis on spatiality, regional differentiation, and complexity in the EEC process in the Yellow River Basin while minimizing the loss of original information

## 4. Results

### 4.1. Spatial Pattern and Spatiotemporal Trends of ESVs

#### 4.1.1. Distribution Pattern of ESVs in the Yellow River Basin

From 2001 to 2015, the total ESV and the ESV per unit area in the Yellow River Basin showed a weak decreasing trend followed by a rapid increase. In 2000, the total ESV was 5291.99 × 10^8^ yuan, and the ESV per unit area was 66.78 × 10^4^ yuan/km^2^. By 2010, the total ESV had decreased to 5256.73 × 10^8^ yuan, and the ESV per unit area had decreased to 66.34 × 10^4^ yuan/km^2^. After 2010, both values increased rapidly. By 2015, the total ESV had increased to 6524.858 × 10^8^ yuan, an increase of 23% over the total value in 2000. The ESV per unit area increased to 82.35 × 10^4^ yuan/km^2^, an increase of 24.13% from its lowest value in 2010. In terms of zoning (Table 2), the total ESVs from 2000 to 2010 in eight provincial regions (not including Ningxia Hui Autonomous Region) showed a decreasing trend, with the largest decline in Sichuan Province (9%). By 2015, the ESVs in all provincial regions had increased rapidly compared with the values in 2000. Based on the ESV analysis per unit area, the three provinces of Sichuan, Shanxi, and Henan had relatively high values per unit area, all of which were greater than 80 × 10^4^ yuan/km^2^. The ESVs per unit area in Ningxia and Inner Mongolia were relatively low. This reflected the gradient difference between geographical locations and natural climatic conditions, which directly affected the surface vegetation cover and directly impacted the ESVs. During the 15 studied years, there was a significant difference in the rate of change in the ESV per unit area. The rates of change in the ESV per unit area in Qinghai and Sichuan were negative, and Sichuan had the largest decline (−13.68%). The remaining seven provincial regions showed positive growth. The largest increase was 84.13% in Shandong, followed by Ningxia (74.89%). The increase in the ESV per unit area mainly happened after 2010, reflecting the positive effects of regional ecological governance and restoration projects with an emphasis on “green” measures.

**Figure 2:**
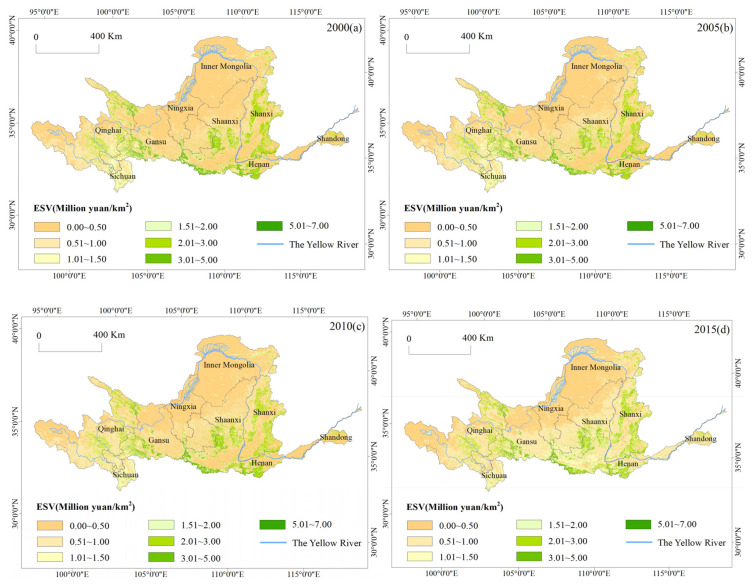
Spatial distribution of ESVs in the Yellow River Basin. (**a**) ESVs in 2000. (**b**) ESVs in 2005. (**c**) ESVs in 2010. (**d**) ESVs in 2015.

The spatial distribution of ESVs in the Yellow River Basin showed high values in the west and southeast and low values in the north (Figure 2). This pattern was highly correlated with the characteristics of vegetation coverage and the distribution of land cover types in the Yellow River Basin. The area with high ESVs was U-shaped and was mainly located west of the Qinghai-Tibet Plateau; in the Qilian Mountains, the Qinling Mountains, the Guanzhong Basin, and the Shanxi Plateau; and southwest of the Taihang Mountains. These areas are mainly mountains and plateaus. Human activities have had little impact in these areas. The terrain is at high elevations, and the amount of precipitation is relatively high. Hence, biomass and vegetation coverage index values are both relatively high [41]. The ESVs of these regions were generally greater than 150 × 10^4^ yuan/km^2^. The areas with low ESVs were large and mainly distributed in the Three Rivers’ Headstream Region, northern Loess Plateau, Ordos Plateau, and western Luliang Mountains. The ESVs in these areas were generally less than 100 × 10^4^ yuan/km^2^. These areas were mainly located in arid and semiarid areas with scarce rainfall, uneven hydroclimate conditions, and severe soil erosion, which significantly affect the growth of living organisms and vegetation. Figure 2 reveals that the range of middle to high ESVs in the central and eastern Loess Plateau areas gradually expanded and became more concentrated, reflecting the significant effects of soil erosion control and the construction of the Three-North Shelter Forest Program on the Loess Plateau.

#### 4.1.2. Spatiotemporal Analysis of the ESVs in the Yellow River Basin

**Figure 3:**
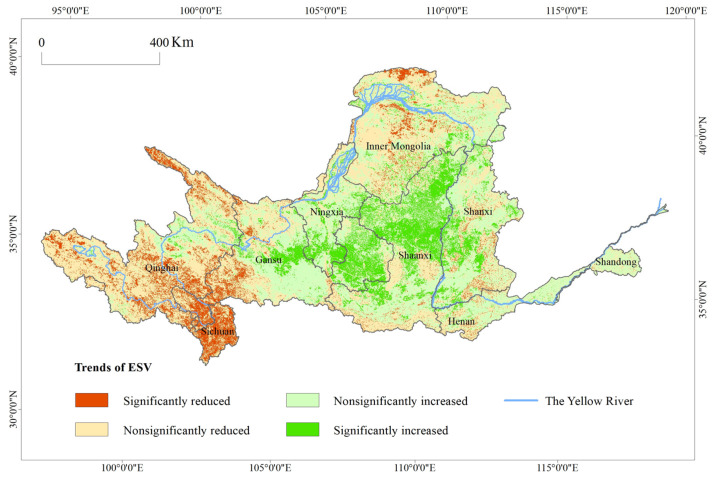
The trends of ESVs in the Yellow River Basin from 2000 to 2015.

It can be seen from Figure 3 that from 2000 to 2015, the spatial variation in the ESVs in the Yellow River Basin was significant, and it had obvious directional and clustering differentiations. The areas with increasing ESVs totaled 44.17 × 10^4^ km^2^, accounting for 55.6% of the river basin’s total area. The areas with significant increases totaled 10.31 × 10^4^ km^2^ and were mainly concentrated in the eastern part of the Yellow River Basin, including the Loess Plateau, southeastern Gansu, central Shaanxi, and western Shanxi. By 2015, the total area of soil and water loss management in the Loess Plateau had exceeded 26 × 10^4^ km^2^ [51]. The forest and grass vegetation coverage had continuously improved, and soil erosion had been effectively controlled. The central and eastern part of the Loess Plateau had become an important growth point for the ESV of the Yellow River Basin. Its agglomeration and diffusion effects will provide a solid foundation and radiating impact on the improvement of the regional ecological environment. The areas with decreasing ESVs were mainly distributed in the Yellow River Basin’s western and northern regions. The areas that had significant decreases totaled 7.27 × 10^4^ km^2^, accounting for 9.1% of the river basin’s total area. They were mainly concentrated in the Three Rivers’ Headstream Region, northern Sichuan, southwestern Qinghai, the Qilian Mountains, and the northern and southern parts of the Hetao Plain. Excessive grazing in the southern Qinghai and the Three Rivers’ Headstream Region caused severe degradation of alpine grasslands. The deforestation in the upper reaches of the Yangtze River led to a continuous decline in forest coverage. Ecological problems, such as illegal mining in the Qilian Mountains, led to the destruction of vegetation [52]. The continued spread of built-up areas made land desertification more severe.

#### 4.1.3. Stability and Continuity of the ESV in the Yellow River Basin

**Figure 4:**
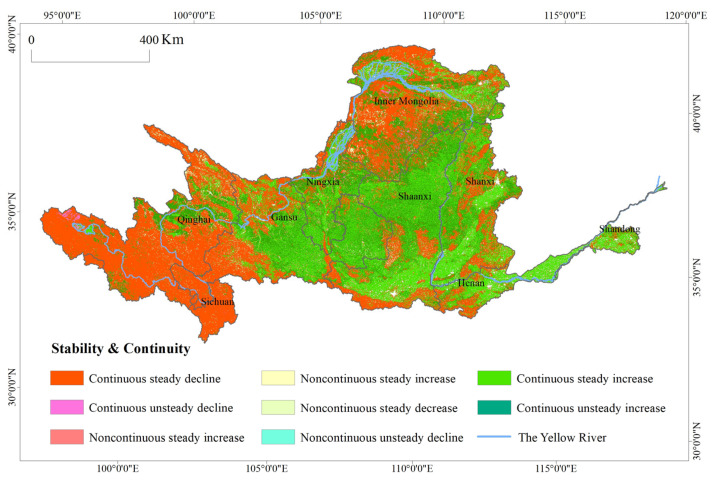
Stability and sustainability of ESVs in the Yellow River Basin from 2000 to 2015.

The analysis above mainly explains the patterns and processes of the ESVs in the Yellow River Basin over the past 15 years, and it remains uncertain what kind of trends will develop in the future. Therefore, based on the stability and continuity model, the future evolution characteristics of the ESV stability and continuity in the Yellow River Basin were analyzed (Figure 4). Figure 4 shows significant spatial and regional differences in the stability and continuity of ESVs in the Yellow River Basin, which show a clustered, compact distribution. A continuous steady decline, a continuous steady increase, and a continuous unsteady increase were the predominant categories. The areas of continuous steady decline were broad, accounting for 41.9% of the study area. They were mainly distributed in the western, northern, and southern edges and showed a pattern of aggregation and clustering. This result could fully reflect the integrity of the ecosystem. The damage of one point source or function could cause a chain reaction and a regional degradation of the ESV. In the ongoing effort to increase the ESV and comprehensive management of the river basin’s ecological environment, the category of continuous steady decline will be the key target of management. Identifying the targets and leaving no gaps will ensure the best policy implementation in the region.

The continuous steady increase areas and the continuous unsteady increase areas made up 25.3% and 26.3% of the study area, respectively. Their distribution patterns were consistent with the previously analyzed ESV trends. This further confirmed the cumulative effects and solid results of the Loess Plateau’s ecological and environmental restoration and provides a model for ecological and environmental governance in other regions in the future. The areas of continuous unsteady increase were mostly attached to the edges of the areas of continuous steady increase. There is a certain amount of uncertainty in the future trends. It is necessary to stabilize the results of the earlier restoration in the future.

In addition, the areas of noncontinuous steady increase and areas of noncontinuous unsteady increase could not be ignored. Their sporadic distribution accounted for approximately 1.47% of the study area. This proportion was relatively small, and the current ESVs showed an upward trend. However, their potential noncontinuous characteristics may become a reality in future development. Therefore, it is necessary to prevent abrupt fundamental changes in the future. In the same way, although areas of noncontinuous steady decline and areas of noncontinuous unsteady decline showed downward trends, their change histories show that they have the potential to show an upward trend. In increasing the ESV, a small investment may produce good results in terms of transformation.

### 4.2. Spatial Pattern of Coordinated Ecological-Economic Development in the Yellow River Basin

**Figure 5:**
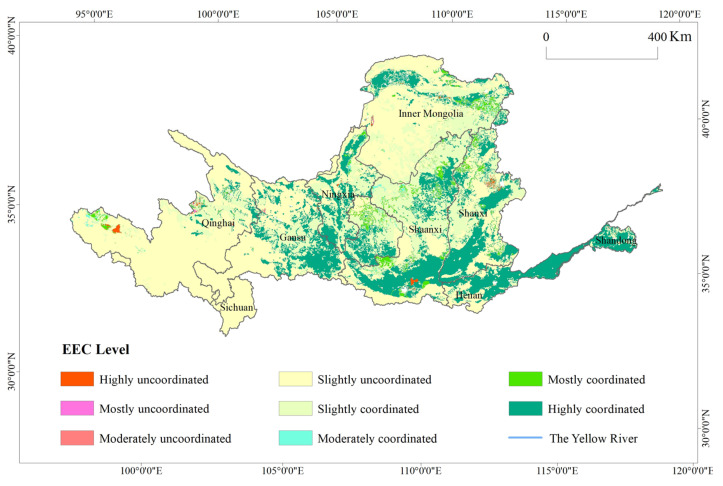
Ecosystem-economic system coordination degree in the Yellow River Basin from 2000 to 2015.

EEC can comprehensively reflect the coordination between ecosystems and the socioeconomic system and can reflect the sustainability of regional economic development. The EEC of the Yellow River Basin was obtained using the EEC model, and the results were divided into eight categories (Figure 5). It can be seen from Figure 5 that the level of EEC in the Yellow River Basin was dominated by slightly uncoordinated areas, followed by slightly coordinated and highly coordinated areas. The areas of the other categories were scattered. The slightly uncoordinated areas had a wide range, accounting for 47.1% of the study area. These areas were mainly distributed in the western Yellow River Basin, Qilian Mountains, the northern side of the Loess Plateau, north of the Hetao Plain, and the southern foot of the Qinling Mountains. These data indicate that the negative externalities of human economic activities had affected the environment. Although the impact was not obvious, the protection of the environment in these areas needs to be strengthened to prevent the issue from getting worse.

The slightly coordinated areas accounted for approximately 25.6% of the study area. They were mainly distributed in the eastern and central parts of the Loess Plateau. The contributions of cultivated land and grassland to the ESVs were high, and the results of years of restoration were prominent. The ESV growth rate was slightly higher than the GDP growth rate. The highly coordinated areas showed a Q-shape and were distributed mostly in the lower and upper portions of the Yellow River Basin, Qinling Mountains, and Guanzhong Basin. These areas represent the critical ecological conservation areas in the Yellow River Basin. Superior hydrologic conditions and geomorphic features provide a good foundation for the growth of the ESV. They are also an essential barrier for ecological security in the river basin.

The mostly and highly uncoordinated areas were mainly urban developed areas, cultivated land, and some water bodies. Urban developed areas were the main places for human economic activities, with intensive human activities and economic output. The growth rate of GDP in these areas was much higher than the growth rate of the ESV. A large amount of cultivated land was claimed during the early urbanization process, resulting in decreases in cultivated land and soil quality and a rapid decline in the ESV. These areas drove economic growth at the expense of the ecological environment, which was an unsustainable state. The reason some water bodies are considered highly uncoordinated areas might be that the unreasonable use of water resources caused the water areas to shrink too much. The decreasing ESVs concealed the stability of the economy, resulting in a highly uncoordinated status.

**Figure 6:**
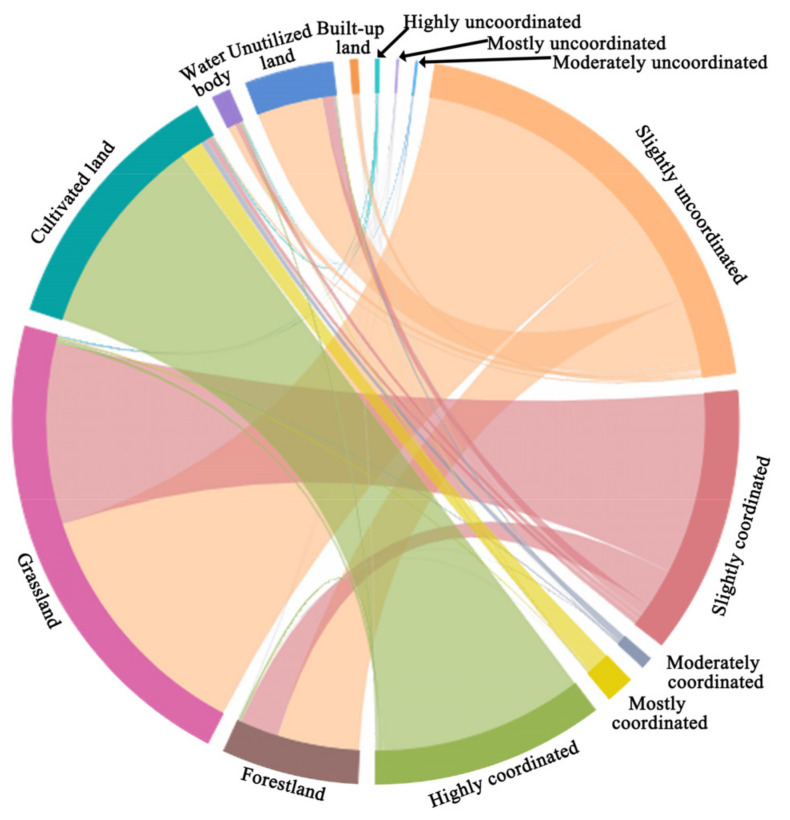
Ecological-economic coordination degree by land use type in the Yellow River Basin.

The initial EEC classification results were superimposed with land use data to explore the level of EEC from the perspective of different land use types (Figure 6). Overall, areas with moderately uncoordinated or worse levels were mainly cultivated land, built-up land, and water areas. Areas with moderately coordinated or better levels occurred mainly in cultivated land, grassland, and forestland. These findings indicate that different land use types led to differences in surface vegetation coverage, which directly impacted the value of the coordination index. The proportion of moderately uncoordinated or worse areas and the proportion of moderately coordinated or better areas were both high in cultivated land, indicating that the results associated with the same land use type under different human activity disturbances could vary greatly. The proportion of all types of uncoordinated areas in forestland was 66.4% from the analysis of different land use types. The largest categories were slightly uncoordinated areas and slightly coordinated areas, which accounted for 66.1% and 31.5% of the total forestland area, respectively.

Mostly uncoordinated areas and moderately uncoordinated areas accounted for smaller proportions. All types of uncoordinated areas made up 57.2% of the grassland, the highest proportion being slightly uncoordinated areas. Among all types of coordinated areas, the proportion of slightly coordinated areas was the highest. In cultivated land, the ratio of coordinated areas was 98.8%, which was much higher than the proportion of uncoordinated areas. Among them, the highly coordinated areas accounted for the largest proportion. In all uncoordinated areas, the slightly uncoordinated areas and the highly uncoordinated areas made up the largest proportions. In all coordinated areas, the slightly coordinated areas and the highly coordinated areas made up the largest proportions. The proportion of uncoordinated areas in waters was basically the same as that of the coordinated areas. The slightly coordinated and slightly uncoordinated areas had sizable proportions. Among the unused land, slightly coordinated areas were the main type and accounted for 82.1% of the total unused land. The areas with moderate coordination or better levels were small. In built-up land, the proportion of uncoordinated areas reached 99.1%. Slightly uncoordinated and moderately uncoordinated areas were the main types. No mostly coordinate areas or highly coordinate areas were found.

## 5. Discussion

In addition to the indirect effects of climate change, human activities have a direct and important impact on land cover and related ecological subsystems [53]. The reasons why ESVs exhibited an initial weak decrease and a later rapid increase were intimately related to the economic development pattern and human economic development intensity of the Yellow River Basin in different periods. Since 2000, urbanization and industrialization in the Yellow River Basin were rapidly implemented [54]. Unsustainable economic processes were pursued at the expense of consuming natural resources and sacrificing the ecological environment. As a result, the overexploitation of natural resources and damage to ecosystem functions caused the ESVs to decline. After 2010, the concepts of an ecological civilization and green development have gradually been integrated into all aspects of development and the entire process of economic and social construction [55]. The structural effects brought about by the technological innovation-led industrial reform gradually manifested, and vigorous implementation of ecological restoration projects, such as afforestation and conversion of farmland to forests and grasslands, has effectively redressed ecological problems in the Yellow River Basin. The ESV continues to grow.

At present, there are few studies that have evaluated the ESV of the whole Yellow River Basin. Compared with the existing studies [54,56], this paper fully considers the differences of ecosystem quality in the calculation of the ESV and, hence, reduces the estimation errors. In addition to climatic conditions, human activities play an important role in changes in the ESV. This conclusion is consistent with the research of Li et al. [57]. With the continuous comprehensive management of the Loess Plateau, the vegetation coverage has improved [58]. Therefore, the Loess Plateau has becoming an area with significantly increasing ESV in the Yellow River Basin. Human activities play a positive role in promoting the healthy development of the ecosystem.

To realize the coordinated development of ecological protection and the economy in the Yellow River Basin, we propose different policy measures for the comprehensive management of the ecological environment of the Yellow River Basin in the future. (1) The overall coordination of the Qinghai-Tibet Plateau was at slightly coordinated or better levels. Therefore, the goal should be to improve the water conservation capacity. The Three Rivers’ Headstream Region, Qilian Mountains, western Longnan, and northern Sichuan are the key areas for wetland conservation, natural forest conservation, and grassland restoration [59,60]. (2) The ecological environment in the Loess Plateau has improved significantly. It is necessary to strengthen further and improve the effectiveness of restoration. Soil erosion control and conversion of farmland to forests and grasslands should be continuously implemented [61]. (3) The North China Plain has a low level of EEC. It is necessary to promote the transformation of economic development methods further and advocate the establishment of a low-carbon green energy economic system to achieve high-quality development. The optimization and upgrading of the Yellow River Basin’s industrial structure should be actively promoted to achieve the goals of industrial ecology and industrialization of the ecological environment. (4) Of all the built-up land, the proportion of uncoordinated areas reached 99.1%. No mostly coordinated areas or highly coordinated areas were found. This also reflects the urgent need to change the existing industrial development model. The upgrading of industrial systems can be initiated from the outlooks of ecological agriculture, ecological industry, and ecotourism [62,63]. Guided by demand, more ecological products will be produced, and the economic and social values of ecological resources will be fully realized.

There were certain limitations in this study. First, the ecosystem contains multiple subsystems and provides multiple service functions. This paper explores nine service functions of six types of ecosystem in the Yellow River Basin and realizes the value estimation of the main service functions of the ecosystem. Due to the lack of data, it is impossible to comprehensively measure the value of every service function in the ecosystem. In particular, the estimations of some non-monetized ecosystem services bring insurmountable difficulties to the accurate calculation of ecosystem service values, leading to inevitable errors in the evaluations. Second, this study took vegetation coverage and NPP as parameters for ecological adjustment. In addition to these, factors such as terrain and climate also have a certain impact on the ESV. The next step is to improve the multivariable ecological adjustment parameter system. Third, the initial value of the unit area of built-up land was set to 0 yuan/km^2^ with reference to existing research results, which ignored the existence of some green space within the urban systems. Green space can also generate a certain amount of ESV, so the calculated result might be smaller than the actual value. Fourth, the ecosystem and the economic systems are the inverse of each other and a unified entity. Complex flows of matter, energy, and information exist in the complex networks of relationships. This study analyzes the state and pattern of EEC from a macroscale perspective. Using only the two indicators of ESV and GDP to represent the two systems may lead to certain limitations at a more detailed level. Therefore, exploring the value conversion and flow of various components on the medium- and microscales in ecosystems and the economic system will be a focus in upcoming research. With the capitalization on ecosystem services and the reform of the property rights system of natural resources, how ESVs can promote the reasonable allocation of resources and ecological and environmental conservation from the aspects of capital and property rights will be valuable for research.

## 6. Conclusions

(1) From 2001 to 2015, the total ESV and ESV per unit area in the Yellow River Basin showed a weak decline followed by a rapid increase. The total ESV increased by 23% in 15 years. The ESV per unit area increased to 82.35 × 10^4^ yuan/km^2^. Qinghai and Sichuan showed decreases in ESV per unit area. The other seven provincial regions all showed increases. The spatial distribution of ESVs showed high values in the west and southeast and low values in the north. In most areas of the Loess Plateau, effective comprehensive management has significantly improved the ESV.
(2) From 2000 to 2015, the spatiotemporal ESV trend in the Yellow River Basin was directional, clustered, and differentiated. The total area showing an upward ESV trend accounted for approximately 55.6% of the river basin’s total area, with the central area of the Loess Plateau as the focal area. The areas showing a downward trend were mainly concentrated in the Yellow River Basin’s western and northern regions. Unsustainable human development activities were the main reason for the significant decline in the ESV.
(3) There were significant spatial and regional differences in ESV’s stability and continuity in the Yellow River Basin, which showed a clustered, compact distribution. The areas of continuous steady decline, continuous steady increase, and continuous unsteady increase made up the largest proportions of the river basin’s total area. Other types were sporadic. The future ecological plans should focus on areas of continuous steady decline, reinforce areas of continuous steady increase, and control areas of noncontinuous steady increase and areas of noncontinuous unsteady increase.
(4) The level of EEC in the Yellow River Basin was dominated by slightly uncoordinated areas, followed by slightly coordinated and highly coordinated areas. The slightly uncoordinated areas were 47.1% of the study area, and the slightly coordinated areas accounted for approximately 25.6% of the study area. The distribution of highly coordinated areas was Q-shaped, mainly distributed in the Yellow River’s lower reaches, Qinling Mountains, and Guanzhong Basin. The level of EEC varied between land use types. The proportion of all types of coordinated areas in the cultivated land area was 98.8%. In built-up land, the proportion of uncoordinated areas was 99.1%.

## Figures and Tables

**Table 1:** The per unit ESVs of different Chinese land ecosystems.

Service type	Ecosystem Service Value/(yuan·km^−2^·a^−1^)					
	Forestland	Grassland	Cultivated Land	Water Body	Unutilized Land	Built-up Land
Gas regulation	30.970	7.079	4.424	0.000	0.000	0.000
Climate regulation	23.891	7.964	7.875	4.070	0.000	0.000
Water conservation	28.315	7.079	5.309	180.332	0.265	0.000
Soil formation and protection	34.509	17.255	12.919	0.088	0.177	0.000
Waste treatment	11.592	11.592	14.512	160.866	0.088	0.000
Biodiversity conservation	28.846	9.645	6.282	22.033	3.008	0.000
Food production	0.885	2.655	8.849	0.885	0.088	0.000
Raw material	23.006	0.442	0.885	0.088	0.000	0.000
Entertainment culture	11.326	0.354	0.088	38.402	0.088	0.000
Total	193.340	64.065	61.143	406.764	3.714	0.000

**Table 2:** ESV changes in the Yellow River Basin (by province) from 2000 to 2015.

Province	2000		2005		2010		2015		ESVper Rate of Change /%
	ESV/ (10^8^ yuan/km^2^)	ESV*_per_*/ (10^4^ yuan/km^2^)	ESV/ (10^8^ yuan/km^2^)	ESV*_per_*/ (10^4^ yuan/km^2^)	ESV/ (10^8^ yuan/km^2^)	ESV*_per_*/ (10^4^ yuan/km^2^)	ESV/ (10^4^ yuan/km^2^)	ESV*_per_*/ (10^4^ yuan/km^2^)	
Qinghai	1153.51	76.70	1174.18	78.07	1140.00	75.80	1094.82	72.79	−5.10%
Sichuan	190.35	102.78	178.05	96.14	172.71	93.26	164.35	88.72	−13.68%
Gansu	1088.31	76.32	1105.04	77.49	1075.53	75.42	1369.87	96.06	25.86%
Ningxia	145.13	28.31	147.37	28.75	167.43	32.66	253.84	49.51	74.89%
Inner Mongolia	433.06	28.72	435.61	28.89	420.05	27.86	524.97	34.82	21.24%
Shaanxi	996.06	75.06	990.45	74.64	1047.82	78.96	1387.17	104.53	39.26%
Shanxi	903.12	93.40	877.93	90.80	867.43	89.712	1144.64	118.40	26.77%
Henan	313.72	86.14	302.77	83.13	301.86	82.88	458.72	125.93	46.19%
Shandong	68.72	52.99	71.78	55.36	63.90	49.28	126.49	97.57	84.13%
